# Combined Treatment of Monopolar and Bipolar Radiofrequency Increases Skin Elasticity by Decreasing the Accumulation of Advanced Glycated End Products in Aged Animal Skin

**DOI:** 10.3390/ijms23062993

**Published:** 2022-03-10

**Authors:** Seyeon Oh, Nark-Kyoung Rho, Kyung-A Byun, Jin Young Yang, Hye Jin Sun, Miran Jang, Donghwan Kang, Kuk Hui Son, Kyunghee Byun

**Affiliations:** 1 Functional Cellular Networks Laboratory, Department of Medicine, Graduate School and Lee Gil Ya Cancer and Diabetes Institute, College of Medicine, Gachon University, Incheon 21999, Korea; seyeon8965@gmail.com (S.O.); kabyun95@gmail.com (K.-A.B.); roswellgirl111@gmail.com (J.Y.Y.); 2 Leaders Aesthetic Laser & Cosmetic Surgery Center, Seoul 06014, Korea; rhonark@hanmail.net; 3 Department of Anatomy & Cell Biology, Gachon University College of Medicine, Incheon 21936, Korea; 4 Jeisys Medical Inc., Seoul 08501, Korea; sunhj@jeisys.com (H.J.S.); lyla@jeisys.com (M.J.); kang@jeisys.com (D.K.); 5 Department of Thoracic and Cardiovascular Surgery, Gachon University Gil Medical Center, Gachon University, Incheon 21565, Korea

**Keywords:** aged skin, radiofrequency, monopolar, bipolar, nuclear factor erythroid 2-related factor 2, glyoxalase, collagen and elastic fiber synthesis

## Abstract

It is well known that skin aging is related to the destruction of collagen and elastin fibers by metalloproteinases (MMPs). Aged fibroblasts have a decreased ability to synthesize collagen and elastin. Nuclear factor erythroid 2-related factor 2 (NRF2) involves glyoxalase (GLO) activation, which inhibits the production of advanced glycated end products (AGE) and the expression of its receptor (RAGE). RAGE increases nuclear transcription factor-kappa B (NF-κB), which upregulates MMPs and decreases skin elasticity. NRF2 also decreases M1 macrophages, which secrete tumor necrosis factor-alpha (TNF-α), thereby decreasing AGE production. It is well known that radiofrequency (RF) decreases skin elasticity by increasing collagen synthesis. We evaluated whether RF increases skin elasticity via NRF2/GLO and whether they decrease AGE and RAGE expression in aged animal skin. We also compared the effects of RF based on the modes (monopolar or bipolar) or the combination used. In aged skin, NRF2, GLO-1, and M2 macrophage expression was decreased, and their expression increased when RF was applied. M1 and TNF-α demonstrated increased expression in the aged skin and decreased expression after RF application. AGE accumulation and RAGE, NF-κB, and MMP2/3/9 expression were increased in the aged skin, and they were decreased by RF. The papillary and reticular fibroblast markers showed decreased expression in young skin and increased expression in aged skin. The densities of collagen and elastin fiber in the aged skin were low, and they were increased by RF. In conclusion, RF leads to increased collagen and elastin fibers by increasing NRF2/GLO-1 and modulating M1/M2 polarization, which leads to decreased AGE and RAGE and, consequently, decreased NF-κB, which eventually slows collagen and elastin destruction. RF also leads to increased collagen and elastin fiber synthesis by increasing papillary and reticular fibroblast expression.

## 1. Introduction

Skin aging is the most common cosmetic problem. It is a complex process that is caused by both intrinsic and extrinsic aging [1,2,3,4]. Intrinsic aging, also called chronological aging, is caused by the passage of time [1,2,3,4]. Extrinsic aging, also known as photoaging, is a result of environmental influences, such as ultraviolet (UV) radiation or smoking [1,2,3,4]. Both of these aging processes have a common pathophysiology, which is that the skin is affected by oxidative stress or increased reactive oxygen species (ROS) [5]. Increased oxidative stress causes DNA damage, nonenzymatic glycosylation, and amino acid racemization, which lead to the increased abnormal cross-linking of collagen fibers and other structural extracellular matrix (ECM) proteins [2,6,7,8,9]. Thus, oxidative glycation, which enhances the accumulation of advanced glycated end products (AGE), also increases during the skin during aging process [10]. Reactive carbonyls, such as glyoxal or methylglyoxal, and increased ROS lead to the glycation of proteins and lead to AGE formation [11,12].

Glyoxal and methylglyoxal compounds are removed by the glyoxalase (GLO) system, which consist of glyoxalase 1 (GLO1) and 2 (GLO2) [13]. During the aging process, GLO expression changes [13,14,15,16,17,18], and their efficiency also decreases [1,2]. As a compensatory mechanism, the GLO1 expression increases in aged skin [19]. However, GLO2 expression is decreased in the photo-exposed aged skin [19]. These results show that the GLO system contributes to protecting the skin from the oxidative stress that occurs during aging [19]. The GLO1 promoter region is composed of several regulatory elements: nuclear transcription factor-kappa B (NF-κB), activator protein-2α (AP-2α), activator protein-1 (AP-1), early gene 2 factor isoform 4 (E2F4), and antioxidant-response element (ARE) [20,21,22]. Since ARE is also a binding site of nuclear factor erythroid 2-related factor 2 (NRF2), the latter activates the GLO1 inducer [23,24,25]. It is also known that tumor necrosis factor-alpha (TNF-α) leads to decreased GLO1 activity, further resulting in caspase-dependent cell death and increased ROS production [26,27]. ROS inhibits GLO1 activity and increases AGE accumulation [28,29,30,31]. GLO1 expression is decreased by the activation of the receptor for AGE (RAGE) [23,32].

Upon the binding of AGE to RAGE, NF-κB is activated, and it sequentially induces various inflammatory signaling pathways [33,34]. NF-κB is one of the main signaling pathways that increases the expression of metalloproteinases (MMPs) in the skin [35]. MMPs are responsible for the degradation of ECM proteins, such as collagen and elastin, and this leads to decreased skin elasticity and to the development of skin wrinkles [36,37,38].

The macrophages that polarize to M1 exhibit high levels of inducible nitric oxide synthase and synthesize proinflammatory cytokines, including TNF-α, interleukin (IL)-1β, and IL-6. M2 macrophages show increased levels of CD206, CD163, arginase 1, and Kruppel-like factor 4 (KLF4), as well as the secretion of anti-inflammatory cytokines, such as IL-10 [39]. Activated NRF2 leads to increased macrophage polarization to M2 and to the decreased synthesis of proinflammatory cytokines [40].

Radiofrequency (RF) irradiation has been used to improve skin tightening by thermal heating [41]. RF has several advantages compared to laser treatment. More widely, RF could be applied to all skin types, as it produces fewer pigmentation changes after treatment than laser treatments do [42].

RF can be applied by means of a two-electrode configuration, including monopolar and bipolar modes [43]. The monopolar mode delivers an electromagnetic current through one active electrode, which transmits the current to a grounding pad [43]. Bipolar devices only deliver the electrical current between the two placed electrodes. The tissue between the two electrodes could be affected by heat, and the penetration depth of the electrical current is about half the distance between the two electrodes [44]. The penetration depth in the bipolar mode delivers more localized and controlled energy distribution and less discomfort and pain than the monopolar mode [45]. One possible mechanism of how RF increases tissue tightening suggests that it increases the dermal temperature to around 65 °C, and this eventually leads to the partial denaturation of collagen by breaking the hydrogen bonds in the triple helix structure of collagen. Collagen fiber denaturation results in immediate collagen contraction and thickening [46,47,48]. The subsequent tightening is generated by a proliferative wound healing process, which promotes collagen synthesis, elastin reorganization, and angiogenesis [43,46,47]. Moreover, our group reported that RF decreased the expression of IL-6 in UV-radiated keratinocyte and animal skin [49]. RF also decreased TNF-α expression UV-radiated mice skin [50].

Even though RF is known to decrease TNF-α expression in UV-radiated skin, it has not been fully revealed whether RF radiation increases skin elasticity in aged skin via increasing NRF2 and decreasing AGE accumulation. We evaluated the effect of RF in increasing NRF2 and GLO by modulating M1/M2 and decreasing TNF-α in an aged animal model. We hypothesized that RF radiation led to increased NRF2, which resulted in decreased M1. Activated NRF2 and decreased M1 led to increased GLO and decreased TNF-α. Those changes led to decreased AGE accumulation and decreased RAGE and eventually led to decreased NF-κB. The latter implies decreased MMP activation, which eventually yields lower collagen destruction and increased skin elasticity in aged skin. We also compared the studied effects of RF by considering each mode (MM: monopolar mode applied twice, MB: the application of monopolar mode and then bipolar mode, BB: bipolar mode applied twice, BM: the application of bipolar mode and then monopolar mode) and different combinations of them.

## 2. Results

### 2.1. RF Radiation Increases pNRF2 and GLO-1

**Figure 1:**
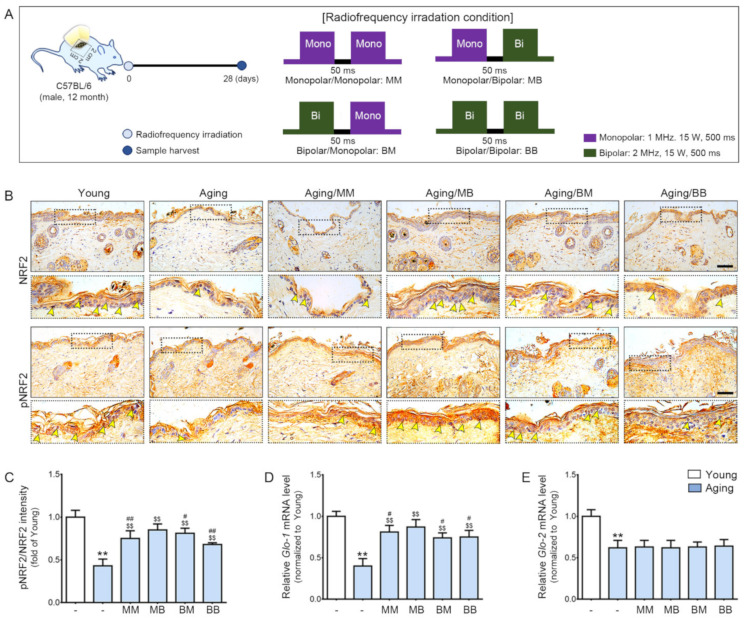
The synergistic effect of pNRF2/NRF2, Glo-1, and Glo-2 expression depending on the combinations of RF modes considered: (**A**) An area of the dorsal skin (2 cm × 2 cm) from the mice in the Young or Aging group without RF radiation or from the mice for each RF mode (MM, MB, BM, or BB) were harvested 28 days after RF radiation. (**B**) NRF2 and pNRF2 expression in the epidermis of the aged mice skin were assessed immunohistochemically (scale bar = 100 µm). The yellow arrows represent positive signals. (**C**) Quantitative ratios of representative NRF2 and pNRF2 images. (**D**,**E**) The mRNA expression levels of (**D**) Glo-1 and (**E**) Glo-2 were determined in the skin tissue. The mRNA levels in the mouse skins were validated by qRT-PCR, normalized versus *Actb*, and expressed relative to levels in the Young group. Data are presented as mean ± standard deviation. **, *p* < 0.01, vs. Young; $$, *p* < 0.01, vs. Aging; #, *p* < 0.05 and ##, *p* < 0.01, vs. Aging/MB (Mann–Whitney *U* test). MM, monopolar mode applied twice; MB, application of the monopolar followed by application of the bipolar mode; BB, bipolar mode applied twice; BM, application of the bipolar mode followed by application of the monopolar mode; Glo, glyoxalase; NRF2, nuclear factor erythroid 2-related factor 2; pNRF2, phosphorylated NRF2.

First, we evaluated the difference between young and aged mice skin. Then, we compared the difference between the aged mice group and the RF-radiated groups by means of the before-mentioned combinations of RF modes. RF was radiated to the dorsal area of old mice after marking the area to receive RF radiation. After 28 days, the skin from the marked dorsal area was harvested. MM is used to represent the group of aged mice on which the monopolar mode was applied twice with a 50 ms interval. MB represents the group of aged mice on which the monopolar mode was applied first, followed by the bipolar one after 50 ms. BB represents the group of aged mice on which the bipolar mode was applied twice with a 50 ms interval. BM represents the group of aged mice on which the bipolar mode was applied first, followed by the monopolar mode (Figure 1A).

The pNRF2 and total NRF2 (pNRF/NRF) ratio was significantly lower in the aged skin, and it was increased by RF radiation of all four modes. The increasing effect was the most prominent in the MB group (Figure 1B,C). Glo-1 expression was significantly lower in the aged skin. RF radiation increased its expression in four modes. The increasing effect was the most prominent in the MB group (Figure 1D). Glo-2 expression was significantly lower in the aged skin (Figure 1E). Unlike Glo-1, the Glo-2 level was not increased by RF.

### 2.2. RF Radiation Modulates M1/M2, and Increases TNF-α, and Decreases IL-10

**Figure 2:**
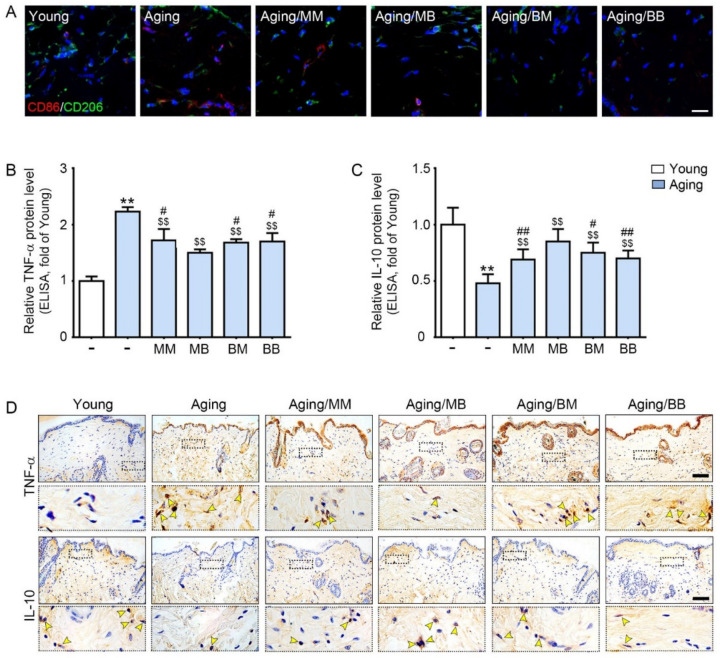
The modulating effect of M1/M2, TNF-α, and IL-10 depending on the considered combinations of RF modes: (**A**) Double-stained confocal microscopic images showing the expression of M1 (CD86, red) and M2 (CD206, green) markers. Nuclei were stained blue using DAPI (scale bar = 100 μm). (**B**,**C**) ELISA was used to determine the protein expression levels of (**B**) TNF-α and (**C**) IL-10 in the skin tissue. (**D**) TNF-α and IL-10 expression in the dermis of the aged mice skin was assessed immunohistochemically (scale bar = 100 µm). The yellow arrows point to positive signals. Data are presented as mean ± SD. **, *p* < 0.01, vs. Young; $$, *p* < 0.01, vs. Aging; #, *p* < 0.05 and ##, *p* < 0.01, vs. Aging/MB (Mann–Whitney *U* test). MM, monopolar mode applied twice; MB, application of monopolar mode followed by application of the bipolar mode; BB, bipolar mode applied twice; BM, application of the bipolar mode followed by application of the monopolar mode; CD86, cluster of differentiation 86; CD206, cluster of differentiation 206; DAPI, 4′,6-diamidino-2-phenylindole; TNF-α, tumor necrosis factor-alpha; IL-10, interleukin-10.

CD86 (an M1 marker, red signals) expression was higher in the skin from the aged mice than it was in the skin from the young mice. RF radiation decreased its expression in all four modes (Figure 2A). CD206 (an M2 marker, green signals) expression was lower in the skin from the aged mice than it was in the skin from the young mice. RF radiation increased its expression in all four modes (Figure 2A).

The mRNA expression of CD86 was significantly higher in the skin from the aged mice than it was in the skin from the young mice. RF radiation decreased its expression in all four modes. The decreasing effect was the most prominent in the MB group (Figure S1A). The mRNA expression of CD206 was significantly lower in the skin from the aged mice than it was in the skin from the young mice. RF radiation significantly increased its expression in all four modes. The increasing effect was the most prominent in the MB group (Figure S1B).

TNF-α and IL-10 expression in the skin were evaluated by both ELISA and tissue staining. Both methods showed that the TNF-α expression was significantly higher in the aged skin. RF radiation decreased its expression in all four modes. The decreasing effect was the most prominent in the MB group (Figure 2B, upper panel of Figure 2D, and Figure S1C). IL-10 expression was significantly lower in the aged skin. RF radiation increased its expression in all four modes. The increasing effect was the most prominent in the MB group (Figure 2C, lower panel of Figure 2D, and Figure S1D).

### 2.3. RF Decreases Accumulation of AGE, Expression of RAGE, and NF-κB in the Aged Skin

**Figure 3:**
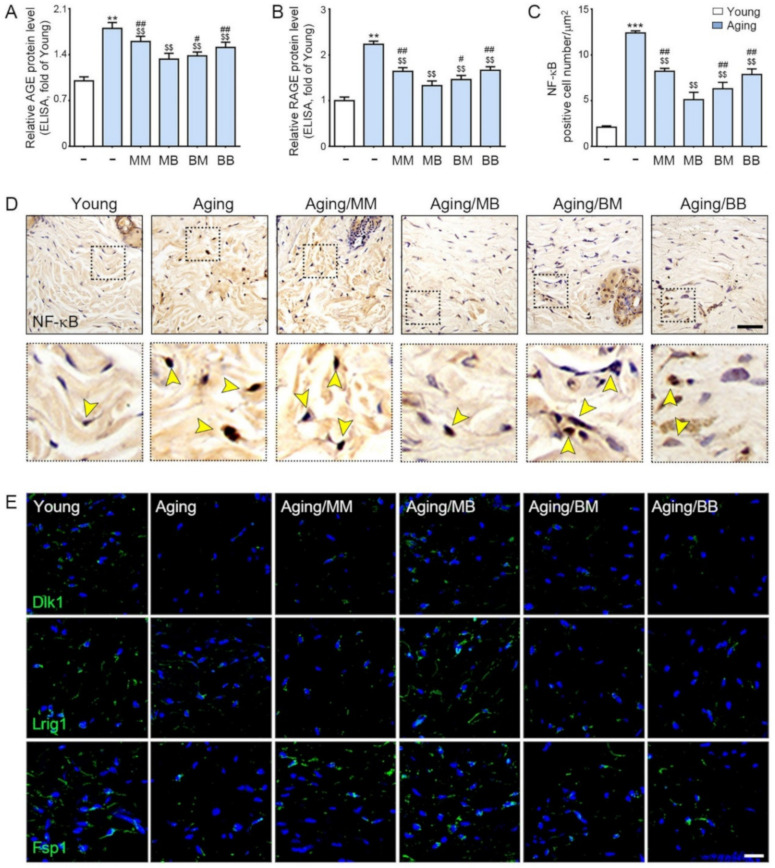
The recovery effect of NF-κB by AGE, RAGE, and papillary and reticular fibroblasts depending on the combination of RF modes: (**A**,**B**) The protein expression levels of (**A**) AGE and (**B**) RAGE were determined in the skin tissue by ELISA. (**C**) Quantitative graphs represent the nuclear NF-κB-positive cell number. (**D**) NF-κB expression in the dermis of the aged mice skin were assessed immunohistochemically (scale bar = 100 µm). The yellow arrows point to positive signals. (**E**) Immunofluorescence analysis showed the expression of Dlk1-, Lrig1-, and Fsp1-positive cells (green). Nuclei were stained blue using DAPI (scale bar = 100 μm). Data are presented as mean ± SD. **, *p* < 0.01 and ***, *p* < 0.001, vs. Young; $$, *p* < 0.01, vs. Aging; #, *p* < 0.05 and ##, *p* < 0.01, vs. Aging/MB (Mann–Whitney *U* test). MM, monopolar mode applied twice; MB, application of monopolar mode followed by application of the bipolar mode; BB, bipolar mode applied twice; BM, application of bipolar mode followed by monopolar mode; AGE, advanced glycated end products; RAGE, rage of advanced glycated end products; NF-κB, nuclear transcription factor-κB; Dlk1, delta-like non-canonical notch ligand 1; Lrig1, leucine-rich repeats and immunoglobulin-like domains 1; Fsp1, ferroptosis suppressor protein 1.

AGE and RAGE were evaluated by ELISA and were determined to be significantly higher in the aged skin. RF radiation decreased their expression in all four modes. The decreasing effect was the most prominent in the MB group (Figure 3A,B). There were significantly more positively stained NF-κB cells in the nuclei in the aged skin. RF radiation decreased NF-κB expression in all four modes. The decreasing effects were the most prominent in the MB group (Figure 3C,D).

### 2.4. RF Increased Expression of Papillary Dermis Fibroblast and Reticular Fibroblast

Delta-like non-canonical notch ligand 1 (Dlk1) is expressed in the dermal fibroblast precursor, reticular fibroblast precursor, and reticular fibroblast [51]. Leucine-rich repeats and immunoglobulin-like domains 1 (Lrig1) are expressed in the dermal fibroblast precursor, papillary dermal fibroblast precursor, and papillary dermal fibroblast [51]. Fibroblast-specific protein 1 (Fsp1) is expressed in the papillary dermal fibroblast [51].

The expression of Dlk1, Lrig1, and Fsp1 was significantly lower in the aged skin than it was in the young skin. RF significantly increased their expression in all four modes. The increase was the most prominent in the MB group (Figure 3E and Figure S2).

### 2.5. RF Decreases MMP2/3/9 and Increases Expression of COL1A1, FBN 1/2, and FBLN 5

**Figure 4:**
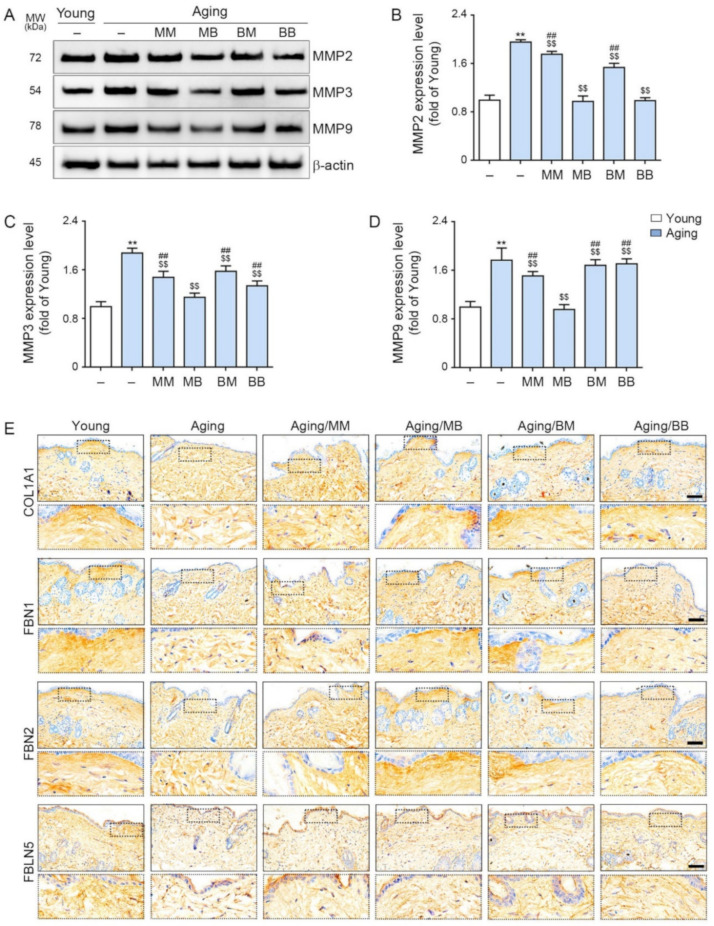
The modulating effect of MMP2/3/9, COL1A1, FBN1/2, and FBLN5 depending on the combinations of RF modes: (**A**) The expression levels of MMP2, 3, and 9 in the skin protein were assessed by Western blot. (**B**–**D**) Quantitative graphs and representative Western blot images. (**E**) COL1A1, FBN1/2, and FBLN5 expression in the dermis of the skin from the aged mice was assessed immunohistochemically (scale bar = 100 µm). Data are presented as mean ± SD. **, *p* < 0.01, vs. Young; $$, *p* < 0.01, vs. Aging; ##, *p* < 0.01, vs. Aging/MB (Mann–Whitney *U* test). MM, monopolar mode applied twice; MB, application of the monopolar mode followed by the bipolar mode; BB, bipolar mode applied twice; BM, application of bipolar followed by monopolar mode; MMP, metalloproteinases; COL1A1, collagen type I α1; FBN, fibrillin; FBLN, fibulin.

MMP2/3/9 expression was significantly higher in the aged skin than it was in the young skin. RF decreased its expression in all four modes. The most prominent decreases were noted in the MB group (Figure 4A–D).

Collagen type I α1 (COL1A1) is the main component of type I collagen [52]. COL1A1 staining determined that COL1A1 expression was significantly lower in the aged skin than it was in the young skin. RF significantly increased its expression in all four modes. The most prominent increases were observed in the MB and BM groups (first row in Figure 4E and Figure S3A).

Fibrillin (FBN) 1 and 2 expression was evaluated by FBN1 and 2 staining and was determined to be significantly lower in the aged skin than in the young skin. RF significantly increased their expression in all four modes. The most prominent increases were observed in the MB group (second and third rows in Figure 4E and Figure S3B,C).

Fibulin (FBLN) 5 was evaluated by FBLN5 staining and determined to be significantly lower in the aged skin than it was in the young skin. RF significantly increased its expression in all four modes. The most prominent increases were exhibited in the MB group (last row in Figure 4E and Figure S3D).

### 2.6. RF Increases Expressions of Collagen and ELASTIN Fiber

**Figure 5:**
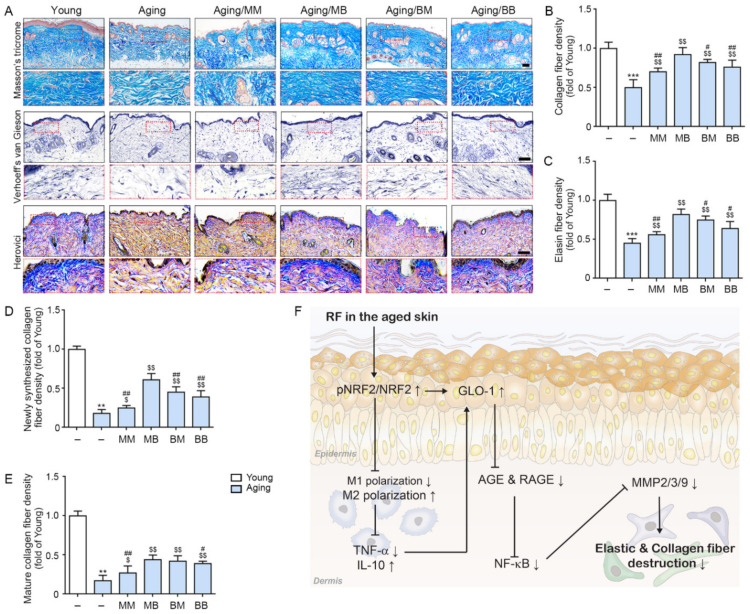
The recovery effect of the collagen and elastin fibers depending on the combinations of RF modes: (**A**) Masson’s trichrome stain (first lane) demonstrated the collagen fibers, Verhoeff’s van Gieson stain (second lane) identified the elastin fibers, and Herovici collagen stain (third lane) demonstrated the amount of young collagen fibers in the dermis of the aged mice skin (scale bar = 100 µm). (**B**–**E**) Quantitative graph representing (**B**) Masson’s trichrome stain, (**C**) Verhoeff’s van Gieson stain images, and (**D**,**E**) Herovici collagen stain images. (**F**) The summary of this study. Data are presented as mean ± SD. **, *p* < 0.01 and ***, *p* < 0.001, vs. Young; $, *p* < 0.05 and $$, *p* < 0.01, vs. Aging; #, *p* < 0.05 ##, *p* < 0.01, vs. Aging/MB (Mann–Whitney *U* test). MM, monopolar mode applied twice; MB, application of monopolar mode followed by bipolar mode; BB, bipolar mode applied twice; BM, application of bipolar mode followed by monopolar mode.

The amount of collagen fiber determined by Masson’s trichrome stain was significantly lower in the aged skin than it was in the young skin. RF significantly increased its expression in all four modes. The most prominent increases were observed in the MB group (first row in Figure 5A,B). Elastin fiber expression was evaluated by Verhoeff’s van Gieson stain and was determined to be significantly lower in the aged skin than it was in the young skin. RF significantly increased its expression in all four modes. The most prominent increases were noted in the MB group (second row in Figure 5A,C). Herovici collagen staining was used to differentiate between newly formed collagen (stained blue) from mature collagen (stained red, third row in Figure 5A) [53,54]. There was significantly less newly formed collagen in the aged skin than in the young skin. RF significantly increased newly formed collagen in all four modes. The most prominent increases were observed in the MB group (Figure 5D). There was significantly less mature collagen in the aged skin than in the young skin. RF significantly increased mature collagen in all four modes. The most prominent increases were observed in the MB group (Figure 5E).

## 3. Discussion

The thickness of dermal collagen decreases, and the collagen bundles in the dermis become thinner and more fragmented during the aging process [55]. The area that is filled with collagen in the papillary dermis decreases to 69.16% in 40-year-old skin and continues to decrease to 45.8% in 100-year-old skin. Unlike the papillary dermis, the area that is filled with collagen in the reticular dermis is stable between 40 and 60 years of age; however, after that point, it continues to decrease up to 100 years of age (58.48% density of collagen bundles) due to cutaneous aging [55]. The elastin fibers also decrease due to aging. The elastic fiber atrophies and the fibrillin-rich microfibrils that form the elastin fibers begin to degrade [56]. Various studies have shown that RF radiation increases skin tightening via collagen fiber contraction and by increasing collagen synthesis [43,46,47].

It is well known that NRF2 expression decreases during the aging process [57,58]. In our study, we evaluated whether RF could upregulate NRF2 and modulate M1/M2 polarization in aged skin. In aged skin, NRF2 expression was lower than it was in young skin. M1 marker expression was higher; however, M2 marker expression was lower in the aged skin than it was in the young animal skin. TNF-α expression was higher, and IL-10 expression was lower in the aged skin than it was in the young skin. RF radiation in the MM, MB, BM, and BB groups led to increased NRF2 and higher M2 and IL-10 expression in the aged skin. However, RF resulted in decreased M1 and TNF-α expression in the aged skin.

It is known that NRF2 leads to increased GLO [25]. Moreover, TNF-α inhibits GLO activity [26,27]. Decreased GLO leads to an increased accumulation of AGE and RAGE expression [33,34]. In our study, *Glo-1* and *Glo-2* expression was lower in the aged skin than it was in the young skin. *Glo-1* expression was increased by RF; however, *Glo-2* expression was not changed by RF. AGE accumulation and RAGE expression in the aged skin were higher than they were in the young skin. RF also contributed to decreasing these expressions.

MMPs, which are upregulated by NF-κB, lead to the destruction of collagen and elastin fibers, causing skin wrinkles [35,37]. In our study, NF-κB and MMP2/3/9 expression was higher in the aged skin than it was in the young skin, and their expression was decreased by RF.

Dermal fibroblasts change during the aging process [59]. It was reported that the number of fibroblasts is lower in old mice than in young ones [60]. Senescent fibroblasts are less proliferative and secrete ECM-degrading proteins and proinflammatory cytokines during the aging process [60]. The fibroblasts in aged skin decrease their ability to produce collagen and elastin fibers [61].

Dermal fibroblasts can be classified in two subsets: papillary and reticular fibroblasts. Papillary fibroblasts exist in the papillary dermis, which is a thin layer of poorly organized collagen fiber bundles [62]. However, reticular fibroblasts exist in the reticular dermis, which is thicker and has a well-organized structure of collagen fiber bundles [62]. Furthermore, papillary and reticular fibroblasts perform different roles during wound healing process. Reticular fibroblasts mediate the initial phase of wound repair by migrating into the wound site and producing a collagen-enriched dermis; however, reticular dermis is unable to form hair follicles [63]. The papillary fibroblasts are present in the wound site at later wound-healing stages and are involved in the re-epithelialization and formation of hair follicles [63].

In our study, we evaluated the expression of the papillary or reticular fibroblasts in aged skin. The markers of papillary and reticular fibroblasts were decreased in the aged skin compared to in the young skin. RF leads to an increase in those markers.

Moreover, COL1A1 expression was lower in the aged skin than in it was in the young skin, and RF increased its expression in our study. Elastin, a major element of elastic fibers, contains tropoelastin. Elastic fibers also contain fibrillin-rich microfibrils, including FBN 1 and FBN 2. The structure that is formed by elastin and fibrillin maintains the skin’s elasticity [64]. Decreasing both elastin and fibrillin leads to decreased skin elasticity [64]. FBLN5, a microfibril component, is an essential ingredient for the formation of elastic fibers [65]. In our study, FBN 1/2 and FBLN 5 expression was lower in the aged skin than it was in the young skin. RF increased the expression of both.

The collagen fiber density, which was evaluated by Masson’s trichrome staining, and the elastin fiber density, which was evaluated by Verhoeff’s van Gieson staining, were also lower in the aged skin than they were in the young skin. RF increased both. Both newly synthesized and mature collagen, which were evaluated using Herovici collagen staining, were decreased in the aged skin and were increased by RF.

In our study, it was observed that RF led to decreased collagen and elastin fiber destruction caused by decreasing MMPs. Moreover, RF led to increased expression of the papillary and reticular fibroblasts, which may result in increased collagen and elastin fiber synthesis. In our study, MB showed a more prominent effect in increasing collagen and elastin fiber density. It is known that monopolar RF penetrate tissue more deeply than bipolar RF do [66]. Thus, we expected that MM would be more effective than MB, BM, and BB because MM could penetrate deeper, stimulating the dermis more than the other combined modes. Contrary to our expectations, the results showed that NRF2/GLO-1 activation and M2 expression were the most prominent in the MB mode. Even though we were not able to decipher the exact mechanism underlying the effectiveness of MB compared to the other combinations of RF modes, we speculated that monopolar RF might produce a preheating of tissue for bipolar RF, enabling it to deliver the current more easily. Since a tissue’s electrical conductivity is a function of the tissue temperature, an increased tissue temperature results in reduced impedance [67].

RF currents are preferentially attracted to areas with reduced impedance [42,68]. Thus, tissue preheating increases its temperature and reduces impedance, creating more favorable conditions through which RF can be delivered [69]. Thus, the monopolar mode was applied before the bipolar one in order to generate heat in the dermis, and the increased temperature caused by monopolar RF led to more current being delivered when the bipolar mode was applied. Moreover, mixing the monopolar and bipolar modes could have a beneficial effect on both the broader and deeper areas of the dermis than MM or BB mode alone. Further studies are necessary to evaluate the exact mechanism underlying the superiority of MB compared to other combinations. However, MB showed that it was the most effective in increasing the expression of the papillary and reticular fibroblasts compared to BM, MM, and BB. Since both papillary and reticular fibroblast function are needed for proper dermis regeneration, we consider MB to be most effective mode for the rejuvenation of aged skin. The mechanism allowing RF to increase papillary and reticular fibroblast expression could not be revealed in our study, thus future studies are needed to evaluate the exact mechanism. Tissue evaluation was only performed once, 28 days after RF. Thus, it is hard to evaluate how the collagen synthesis process undertaken by the fibroblast changes depending on time after RF in the present study. To evaluate the more exact mechanism of how RF affects the papillary or reticular fibroblasts, future studies should evaluate performance at various time points after RF.

Our study showed that RF might improve skin elasticity by decreasing the collagen and elastin fiber destruction caused by MMPs in aged skin. RF leads to increased NRF2 and the modulation of M1/M2 polarization, inducing increased GLO-1 expression and decreased TNF-α expression. These lead to a decreased AGE accumulation and RAGE expression, eventually contributing to decreased NF-κB expression. The latter causes decreased MMPs, which reduce the destruction of collagen and elastin fibers (Figure 5F).

## 4. Materials and Methods

### 4.1. In Vivo model and RF Irradiation

Six-week-old male and female C57BL/6 mice were obtained from Orient Bio. (Seoul, Korea). After an adaptation period of 1 week, males and females were mated one-to-one, and the resulting mouse pups were taken care of for 4 months or 12 months.

The raised 4-month-old male mice were grouped into the Young group (*n* = 3), and the 12-month-old male mice were randomly divided into five groups (*n* = 3 per group) as follows [70]:

(1) Aging (Coupling fluid was applied without RF irradiation)
(2) Aging/MM (Coupling fluid was applied to the mice, and the monopolar mode was applied twice with a 50 ms interval)
(3) Aging/MB (Coupling fluid was applied to the mice, and the monopolar mode was applied followed by the bipolar mode after a 50 ms interval)
(4) Aging/BM (Coupling fluid was applied to the mice, and the bipolar mode was applied followed by the monopolar mode after a 50 ms interval)
(5) Aging/BB (Coupling fluid was applied to the mice, and the bipolar mode was applied twice with a 50 ms interval)

After anesthesia inhalational using isoflurane (HANA Pharm Co., LTD., Seoul, Korea), a section of the dorsal skin that was approximately 2 cm × 2 cm in size was shaved from the mice. Then, the mice were placed on a negative plate and were coated with coupling fluid (Jeisys Medical Inc., Seoul, Korea) in order to increase thermal conductivity on the skin. In the monopolar mode, the RF was irradiated at 1 MHz and 15 W for 500 ms, and in the bipolar mode, they were irradiated at 2 MHz and 15 W for 500 ms. The skin tissues of the sacrificed mice were collected 27 days after RF irradiation (Figure 1A).

The mice were housed in cages with a controlled temperature (23 °C) and a 12 h light/dark cycle with free access to food and water. This study was approved by the Center of Animal Care and Use Ethical Board of the Gachon University (Approval Number LCDI-2021-0066) and was carried out in accordance with the Institutional Animal Care and Use Committee.

### 4.2. RF Irradiation System

The POTENZA^®^ (Jeisys Medical Inc.) is able to carry out impedance checking and has a feedback system. It was used to determine the compensation value by automatically measuring impedance, and RF was emitted using invasive and non-invasive electrode tips. A Diamond tip was used in this study. It is able to sequentially irradiate bipolar and monopolar mode in one shot. Each mode could be adjusted in terms of watts, pulse duration, and hertz. The tip consisted of 16 ea (4 × 4), and non-invasive electrodes that were 2 mm in diameter were arranged at intervals of 2 mm. The tip was approved by NAMSA (Northwood, OH, USA) after biological compatibility testing.

### 4.3. Sample Preparation

#### 4.3.1. Paraffin-Embedded Tissue Sectioning

The skin tissues that were fixed by 4% paraformaldehyde (Sigma-Aldrich, St. Louis, MO, USA) were washed for 30 min for embedding preparation. Skin paraffin blocks made using a tissue processor (Thermo Fisher Scientific, Waltham, MA, USA) were sectioned at 7 µm using a microtome (Leica, Wetzlar, Germany) and were cooked at 37 °C overnight to keep them attached to the slides. The sectioned slides were passed through xylene and four concentrations of ethanol (100%, 95%, 80%, and 70%) to deparaffinate them for staining.

#### 4.3.2. Extraction of RNA and cDNA Synthesis

The frozen skin tissues were ground and homogenized by the RNAiso Plus reagent (Takara, Shiga, Japan) according to the manufacturer’s instructions. The extracted RNA was quantified by the NanoDrop 2000 spectrophotometer (Thermo Fisher Scientific, Waltham, MA, USA) and was converted to cDNA using a Prime-Script 1ST strand cDNA Synthesis Kit (Takara, Shiga, Japan) for quantitative real-time polymerase chain reaction (qRT-PCR).

#### 4.3.3. Isolation of Protein

The frozen skin tissues were lysed using RIPA buffer (EzRIPA, ATTO, Tokyo, Japan) with proteinase and phosphatase inhibitors. The lysed skin tissues were sonicated and then centrifuged at 14,000× *g* for 15 min at 4 °C. After centrifugation, the isolated protein (supernatant liquids) was aliquoted and quantified by a bicinchoninic acid assay kit (Thermo Fisher Scientific, Inc., Waltham, MA, USA).

### 4.4. Tissue Immunofluorescence

The deparaffinated skin tissue sections were incubated in blocking solution with normal serum or with a mouse-on-mouse detection kit (Vector Laboratories Inc., Burlingame, CA, USA) to block non-specific antigen and antibody binding and were then incubated with antibodies (Table S1) overnight at 4 °C, rinsed with PBS twice, incubated with fluorescent conjugated secondary antibodies for 1 h, and rewashed with PBS. Nuclei were stained with 4′6-diamino-2-phenylindole (DAPI, Sigma-Aldrich) at room temperature for 10 s, and coverslips were mounted using Vectashield mounting medium (Vector Laboratories Inc.). Fluorescence was detected by confocal microscopy (LSM 710, Carl Zeiss, Oberkochen, Germany) and analyzed using Image J software (NIH, Bethesda, MD, USA).

### 4.5. 3,3-Diaminobenzidine Staining

The sectioned skin tissue slides were incubated in 3% hydrogen peroxide in methanol for 30 min at room temperature to block endogenous peroxidase. The tissue slides were washed using a phosphate-buffered saline (PBS) and then incubated with primary antibodies (Table S1) in normal serum for 24 h at 4 °C. The slides were rinsed with PBS and incubated with a biotinylated secondary antibody using the ABC kit (Vector Laboratories Inc., Burlingame, CA, USA) for 2 h at room temperature. After washing with PBS, the tissue slides were developed over the course of 15 min with 3,3′-diaminobenzidine (Sigma-Aldrich) to confirm the brown signal. To identify the nuclei, the tissue slides were stained in hematoxylin solution for 1 min and then mounted with dibutyl phthalate polystyrene xylene mounting solution (Sigma-Aldrich). Images of the stained tissues were taken under an optical microscope (Olympus Optical Co., Tokyo, Japan) and analyzed using ImageJ software (NIH, Bethesda, MD, USA) [71].

### 4.6. Quantitative Real-Time Polymerase Chain Reaction

For qRT-PCR, a mixed reagent containing SYBR Green reagent (Takara), 1 µg of synthesized cDNA template, and a 10-pmol primer (Table S2), was dispensed into 384-well multiplates, and then analyzed by the CFX386 Touch Real-Time PCR System (Bio-Rad, Hercules, CA, USA).

### 4.7. Enzyme-Linked Immunosorbent Assay (ELISA)

To measure the TNF-α, IL-10, AGE, and RAGE level in the skin, 96-well microplates were coated with 100 nM carbonate and bicarbonate-mixed buffer, adjusted to pH 9.6, and incubated overnight at 4 °C. The microplates were then washed with PBS containing 0.1% Triton X-100 (TPBS). The remaining protein-binding sites were then blocked using 5% skim milk for 6 h at room temperature. After washing with PBS, equal amounts of isolated skin protein samples were distributed to each well and incubated overnight at 4 °C. Each well was rinsed with TPBS and then incubated with primary antibodies (Table S1) diluted in PBS overnight at 4 °C. After washing, a peroxidase-conjugated secondary antibody was loaded for 4 h at room temperature. Tetramethyl benzidine solution was added, followed by incubation for 15–20 min at room temperature. Sulfuric acid (2N) was used as a stop solution. The optical density was measured at a wavelength of 450 nm using a microplate reader (Spectra Max Plus; Molecular Devices, San Jose, CA, USA). 

### 4.8. Western Blotting

Equal amounts of isolated skin proteins were separated on 8–12% polyacrylamide gels and transferred to polyvinylidene fluoride membranes (Millipore, Burlington, MA, USA) via a power station (ATTO, Osaka, Japan). After blocking with 5% skim milk and washing with Tris-buffered saline with 0.1% Tween 20 (TTBS), the membranes were incubated with primary antibodies (Table S1) at 4 °C and then washed with TTBS. The membranes were then incubated with a secondary antibody (Vector Laboratories, Burlingame, CA, USA) and rinsed with TTBS. Subsequently, an enhanced chemiluminescence detection reagent (GE Healthcare, Chicago, IL, USA) was used to visualize the immunoreactive proteins on the membrane.

### 4.9. Histological Analysis

#### 4.9.1. Masson’s Trichrome Staining

After deparaffination, the skin tissues were incubated in Bouin solution (ScyTek, TRM-2; West Logan, UT, USA) for 1 h at 60 °C, rinsed with distilled water, and then incubated in a working weight’ solution of iron hematoxylin for 5 min, a Biebrich scarlet acid fuchsin solution for 5 min, a phosphomolybdic-phosphotungstic acid solution for 12 min, and an aniline blue solution for 3 min. Afterward, the tissues were washed in distilled water, dehydrated in absolute alcohol, and mounted for observation. The collagen fiber densities were measured using ImageJ software [72].

#### 4.9.2. Verhoeff’s van Gieson Staining

The deparaffinated skin tissue slides were incubated in working elastic stain solution (ScyTek, ETS-1, West Logan, UT, USA) for 15 min at room temperature, rinsed three times with tap water, and then incubated in 20 drops of 2% ferric chloride differentiation solution. Afterward, the tissues were washed in distilled water, dehydrated in absolute alcohol, and mounted for observation. The elastin fiber densities were measured using the ImageJ software [73].

#### 4.9.3. Herovici Collagen Staining

We used the Herovici collagen stain kit (Scytek, HSK-IFU) to differentiate between mature collagen (red) and young collagen or reticulin (blue) [53,54]. The deparaffinated slides were incubated for 8 min in Weigert’s Iron Hematoxylin to stain nuclei. Then, the slides were rinsed in tap water for 2 min and then again in distilled water. Then, the washed slides were incubated for 2 min in Herovici solution. Afterward, the stained slide was rinsed with absolute alcohol, dehydrated with absolute alcohol, and mounted using xylene and DPX for observation. The newly synthesized collagen fiber and mature collagen densities were measured using ImageJ software [74].

### 4.10. Statistical Analysis

We performed a Kruskal–Wallis test to compare the six groups, followed by a Mann–Whitney *U* test as a post hoc test. This study was validated using an unpaired *t*-test carried out using SPSS version 22 (IBM Corporation; Armonk, NY, USA). All the results are presented as mean ± standard deviation, and all the experiments were repeated in triplicate. The statistical significance was displayed: *, vs. Young; $, vs. Aging; #, vs. Aging/MB.

## Data Availability

All data are contained within the article.

## Supplementary Materials

The following are available online at https://www.mdpi.com/article/10.3390/ijms23062993/s1.
