# Supplementary material for: Combined Treatment of Monopolar and Bipolar Radiofrequency Increases Skin Elasticity by Decreasing the Accumulation of Advanced Glycated End Products in Aged Animal Skin
Source: Int J Mol Sci. 2022 Mar 10;23(6):2993. doi: 10.3390/ijms23062993 (PMC8950306; doi:10.3390/ijms23062993)
Supplement: Supplementary file 1 [file ijms-23-02993-s001.zip › ijms-1478832-Supplementary.pdf]

Supplementary Tables

Table S1: List of antibodies for immunohistochemistry (IHC), immunofluorescence (IF), ELISA and western blot (WB).

Antigen (host)	Company	Catalog no.	Dilution rate
NRF2 (Rabbit)	Bioss	BS-1074R	1:200 (IHC)
pNRF2 (Rabbit)	Bioss	BS-2013R	1:200 (IHC)
CD86 (Mouse)	Santa cruz biotechnology	sc-19617	1:50 (IF)
CD206 (Rabbit)	Novus Biologicals	NBP1-90020	1:500 (IF)
TNF-α (Mouse)	Novus Biologicals	NBP1-19532	1:50 (ELISA)<br>1:200 (IHC)
IL-10 (Rabbit)	Santa cruz biotechnology	sc-8438	1:50 (ELISA)<br>1:50 (IHC)
AGE (Rabbit)	abcam	ab23722	1:1,000 (ELISA)
RAGE (Mouse)	Santa cruz biotechnology	sc-365154	1:200 (ELISA)
NF-κB (Rabbit)	Cell signaling technology	8242	1:200 (IHC)
Dlk1 (Mouse)	Santa cruz biotechnology	sc-376755	1:50 (IF)
Lrig1 (Mouse)	Santa cruz biotechnology	sc-514577	1:50 (IF)
Fsp1 (Rabbit)	Biorbyt	orb88159	1:100 (IF)
MMP2 (Rabbit)	LSBio	LS-C352523	1:500 (WB)
MMP3 (Rabbit)	CUSABIO technology	CSB-PA17509A0Rb	1:500 (WB)
MMP9 (Rabbit)	GeneTex	GTX31891	1:500 (WB)
β-actin (Rabbit)	Cell signaling technology	4967	1:1000 (WB)
COL1A1 (Mouse)	Santa cruz biotechnology	sc-293182	1:50 (IHC)
FBN 1 (Mouse)	GeneTex	GTX23090	1:100 (IHC)
FBN 2 (Mouse)	Santa cruz biotechnology	sc-393968	1:50 (IHC)
FBLN 5 (Rabbit)	Proteintech	12188-1-AP	1:200 (IHC)

Table S2. List of primers for qRT-PCR used in this study.

Gene		Primers
Glo-1	Forward	5′-CCT GCT ATG AAG TTC TCG CTC T-3′
	Reverse	5′-CTG TCT TCT CGG ACT TGT CCT T-3′
Glo-2	Forward	5′-TAT GAG GGA ACT GCA GAT GAG A-3′
	Reverse	5′-GAC TTT TGT GTC TGG AGG AAG C-3′
CD86	Forward	5′-TTG CTG ATC TCA GAT GCT GTT T-3′
	Reverse	5′-AGG CTT ATG TTT TGA GCC TTT G-3′
CD206	Forward	5′-GAA CCC ATT TAT CAT TCC CTC A-3′
	Reverse	5′-TCC CTA TGG ATC CTG TAG CAG T-3′
Blimp1	Forward	5'-ATG GAG GAC GCT GAT ATG ACT T-3′
	Reverse	5'-GGG GTG GTC GTT CAC TAT GTA T-3′
Dlk1	Forward	5′-CAT CGT CTT TCT CAA CAA GTG C-3′
	Reverse	5′-TGC AAC AGG AGG TTC TTC TTC T-3′
Lrig1	Forward	5′-TCT CCA CAC CAT CCT TAC AGT G-3′
	Reverse	5′-TGT CAT GGT TGC TTG GAT AGA G-3′
Fsp1	Forward	5′-TGT AAT TGT GTC CAC CTT CCA C-3′
	Reverse	5′-AGC TCC TTG AGC TCT GTC TTG T-3′
Actb	Forward	5'-CCG TAA AGA CCT CTA TGC CAA C-3′
	Reverse	5'-GCA GTA ATC TCC TTC TGC ATC C-3′

Supplementary Figures

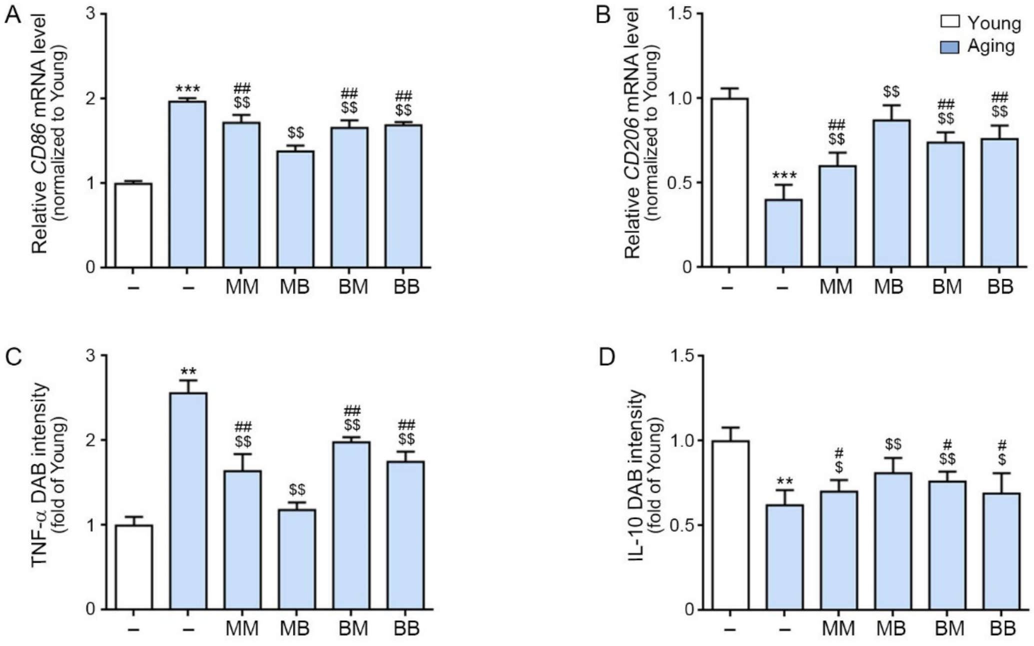

Figure S1. The regulatory effect of M1 and M2 depending on the considered combinations of RF modes. (**A and B**) The mRNA expression levels of (**A**) CD80 (M1 marker) and (**B**) CD206 (M2 marker) were determined in the skin tissue. The mRNA levels in the mouse skins were validated by qRT-PCR, normalized versus *Actb*, and expressed relative to levels in the Young group. (**C and D**) Quantitative graphs of representative TNF-α and IL-10 images of Figure 2D. Data are presented as mean ± standard deviation. **, $p < 0.01$, and ***, $p < 0.001$ vs. Young; $$, $p < 0.01$, vs. Aging; #, $p < 0.05$ and ##, $p < 0.01$, vs. Aging/MB (Mann–Whitney U test). MM; twice apply of monopolar mode, MB; monopolar then bipolar apply, BB; twice apply of bipolar mode, BM; bipolar then monopolar apply; CD80, Cluster of differentiation 80; CD206, Cluster of Differentiation 206; TNF-α, tumor necrosis factor-alpha; IL-10, Interleukin-10.

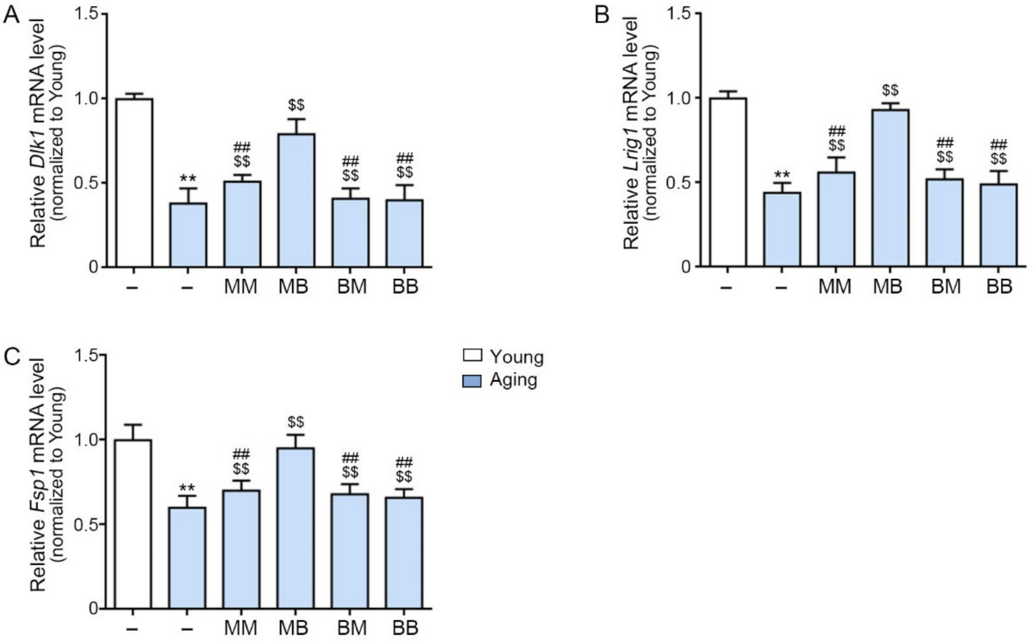

Figure S2. The recovering effect of papillary and reticular fibroblasts depending on the considered combinations of RF modes. (**A–C**) The mRNA expression levels of (**A**) Dlk1 (dermal fibroblast precursor marker), (**B**) Lrig1 (dermal fibroblast precursor, papillary dermal fibroblast precursor, and papillary dermal fibroblast marker) (**C**) Fsp1 (papillary dermal fibroblast marker) were determined in the skin tissue. The mRNA levels in the mouse skins were validated by qRT-PCR, normalized versus *Actb*, and expressed relative to levels in the Young group. Data are presented as mean ± SD. **, $p < 0.01$, vs. Young; $$, $p < 0.01$, vs. Aging; ##, $p < 0.01$, vs. Aging/MB (Mann–Whitney U test). MM; twice apply of monopolar mode, MB; monopolar then bipolar apply, BB; twice apply of bipolar mode, BM; bipolar then monopolar apply; Dlk1, Delta-like non-canonical notch ligand 1; Lrig1, Leucine-rich repeats and immunoglobulin-like domains 1; Fsp1, ferroptosis suppressor protein 1.

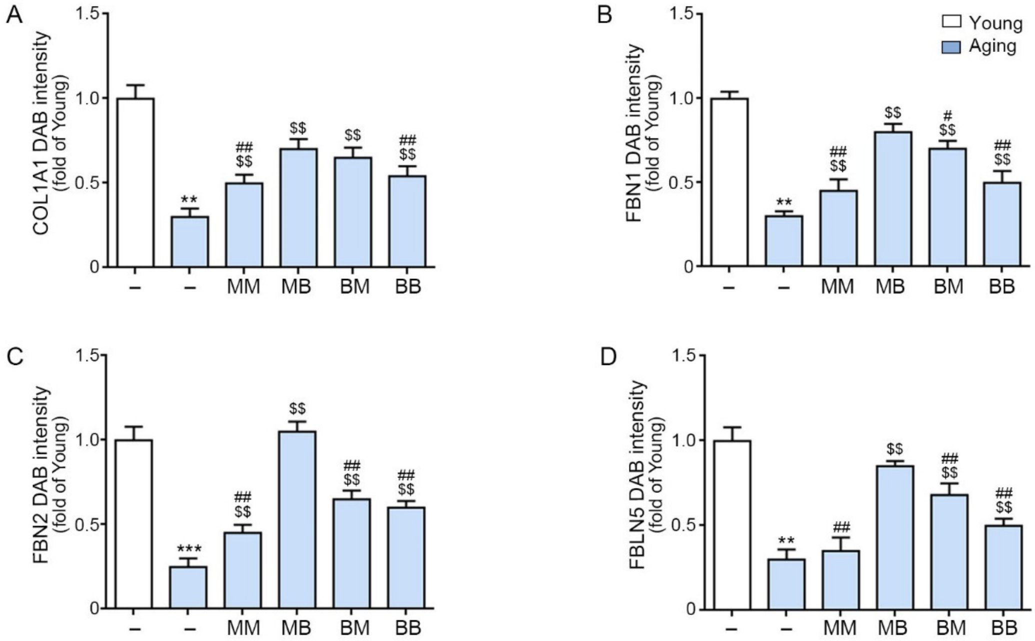

Figure S3. The recovering effect of the COL1A1, FBN1/2, and FBLN5 depending on the combinations of RF modes. (**A**–**D**) Quantitative graph of representative (**A**) COL1A1, (**B**) FBN1, (**C**) FBN2, and (**D**) FBLN5 images of Figure 4E. Data are presented as mean ± SD. **, $p < 0.01$, vs. Young; $$, $p < 0.01$, vs. Aging; #, $p < 0.05$ or $p < 0.01$, vs. Aging/MB (Mann–Whitney U test). MM; twice apply of monopolar mode, MB; monopolar then bipolar apply, BB; twice apply of bipolar mode, BM; bipolar then monopolar apply; COL1A1, Collagen type I $\alpha1$; FBN, fibrillin; FBLN, fibulin.
